# Diverse Anelloviruses Identified in Leporids from Arizona (USA)

**DOI:** 10.3390/v17020280

**Published:** 2025-02-18

**Authors:** Matthew D. De Koch, Nicholas Sweeney, Jesse E. Taylor, Fletcher Lucas, Nichith K. Ratheesh, Stephanie K. Lamb, Janice Miller, Simona Kraberger, Arvind Varsani

**Affiliations:** 1 The Biodesign Center for Fundamental and Applied Microbiomics, Center for Evolution and Medicine, Arizona State University, Tempe, AZ 85287, USA; 2 School of Life Sciences, Arizona State University, Tempe, AZ 85287, USA; 3 Liberty Wildlife, 2600 E Elwood St, Phoenix, AZ 85040, USA; 4 Structural Biology Research Unit, Department of Integrative Biomedical Sciences, University of Cape Town, Rondebosch, Cape Town 7700, South Africa

**Keywords:** *Anelloviridae*, *Lepus californicus*, *Sylvilagus audubonii*, *Dermacentor parumapertus*

## Abstract

The communities of viruses studied in rabbits and hares (family Leporidae) have largely been those with clinical significance. Consequently, less is known broadly about other leporid viruses. Anelloviruses (family *Anelloviridae*) are likely commensal members of the single-stranded DNA virome in mammals. Here, we employ a viral metagenomic approach to identify DNA viruses of leporids and the ticks feeding on them in Arizona, USA. We characterize five complete anellovirus genomes from four leporids belonging to the black-tailed jackrabbit (*Lepus californicus*, n = 3) and the desert cottontail (*Sylvilagus audubonii*, n = 1). All five anellovirus genomes share > 69% *orf1* gene pairwise nucleotide identity with those found in Iberian hares and thus belong to the species *Aleptorquevirus lepor1*. Accordingly, we expand the known host range of this anellovirus species to include Iberian hares in Europe and black-tailed jackrabbit and desert cottontail in the USA. We also sequenced the complete mitochondrial genomes of the four leporid hosts (*Sylvilagus audubonii*, n = 1; *Lepus californicus*, n = 3) and two ticks (*Dermacentor parumapertus*, n = 2) found feeding on two black-tailed jackrabbits. These results expand the diversity of anelloviruses in leporids while giving insight into the host genetics of leporids and ticks in Arizona, USA.

## 1. Introduction

Anelloviruses are negative-sense single-stranded (ss) circular DNA viruses that have been identified in various mammal and avian hosts. The family *Anelloviridae* currently comprises ~150 species classified into ~30 genera, with the majority of hosts of these viruses being mammals [1,2,3,4]. Encapsidated by icosahedral virions, the genomes range in length from 1.6 to 3.9 kb and typically encode at least three open reading frames (ORFs) [5]. Their functional properties have been predicted via structural modeling, yet their precise replicative mechanisms remain unknown. The ORF1 is associated with ssDNA packaging [5] and encodes the capsid protein [6]. The smaller ORF2 and ORF3 proteins may be involved in genome replication and gene expression [7]. Metagenomic studies continue to expand the diverse anellovirus taxonomy, as well as factors shaping ecology and evolution.

Anelloviruses have a ubiquitous presence in various mammalian hosts, with no disease association, thus alluding to a commensal anellovirus–host relationship [7,8,9]. They have varied prevalence within a population; for example, in human populations, this can range from 5% to 90% [10]. In humans, it is hypothesized that infection and acquisition of anelloviruses likely occur shortly after birth, through fecal–oral or airway routes [11,12,13]. Anelloviruses appear to be more generalists with regard to tissue tropism and have been identified from many sample types, including whole blood, saliva, cerebrospinal fluid, and various body tissue types including the brain and feces [14,15,16,17,18,19,20]. Since infection is ubiquitous and does not correlate with disease outcomes, it has been hypothesized that anelloviruses may benefit their hosts [7,9].

In leporids, diverse anelloviruses have been identified in Iberian hares (*Lepus granatensis*) from Spain as coinfection with a recombinant strain of myxoma virus-MYXV-Tol [21] and/or a polyomavirus [22]. There are nine non-coding fragments (~250 nts) of the anellovirus sequence (MZ543316–MZ543324) from *Lepus europaeus* available in GenBank, with a small region (~30 nts) that shares similarity to porcine anelloviruses. Other than this, there is no information on anelloviruses in any other members of the order Lagomorpha. The literature associated with leporid-infecting viruses is largely dominated by those with high pathogenicity, e.g., rabbit hemorrhagic disease virus (family *Caliciviridae*) and myxoma virus (family *Poxviridae*). A recent study on rabbits and their associated ectoparasites identified a large number of novel RNA viruses in the ectoparasites and a few vertebrate-infecting viruses in the families *Caliciviridae*, *Picobirnaviridae*, and *Picornaviridae* [23]. On the other hand, studies by Ning, et al. [24] and Xiao, et al. [25], which focused on the viromes of laboratory rabbits, identified viruses in the families *Circoviridae*, *Coronaviridae*, *Parvoviridae*, *Polyomaviridae*, and *Picobirnaviridae* from blood, feces, skin, and oral samples.

**Figure 1:**
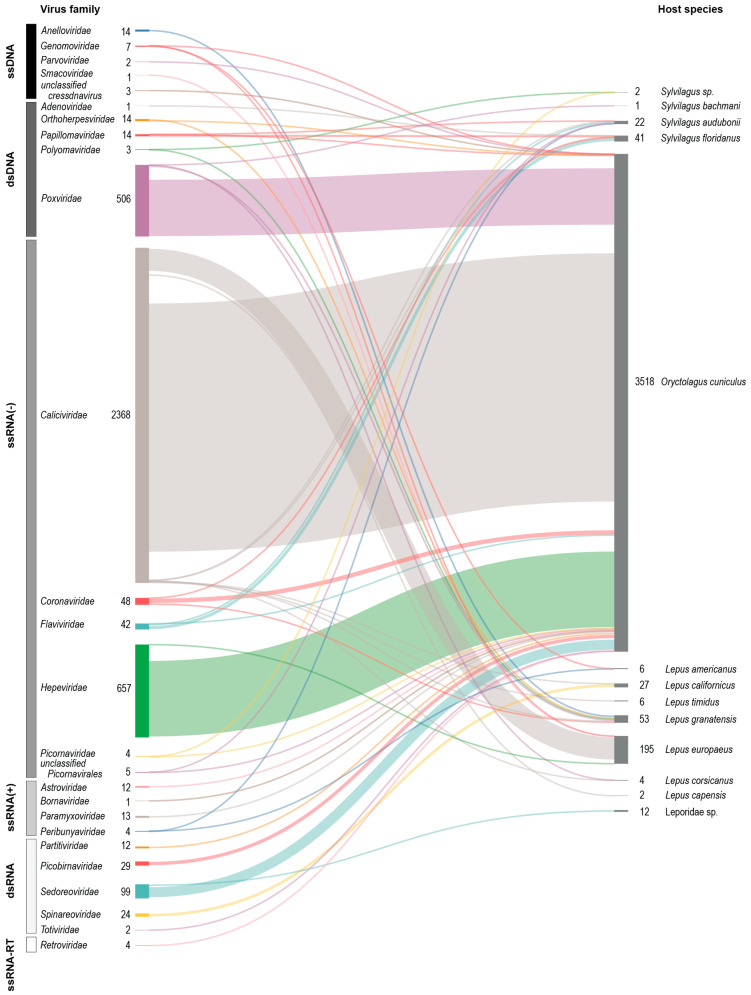
Virus sequences from various leporids. Summary of the virus sequences from leporids based on sequence data available in NCBI Virus [26]. The Sankey plot was generated using SankeyMATIC (https://github.com/nowthis/sankeymatic, accessed on 27 November 2024) [32] and shows the number of sequences of viruses in various families and their source /host.

Thus, as a result, sequence databases are equally skewed based on more efforts being concentrated on more clinically relevant and pathogenic viruses [26]. An overview of leporids based on the sequence data available in NCBI Virus is shown in Figure 1.

Anelloviruses have previously been identified in ticks [27,28] and mosquitos [29], likely from feeding on vertebrate hosts. Arthropod vectors carry several important pathogens infecting humans and animals. The parasitic nature of ticks as obligate blood-feeders facilitates the transmission of pathogens with their vertebrate hosts. Among arthropods, high-throughput sequencing approaches have also facilitated both RNA and DNA virus discovery [30,31]. However, similar to the viruses in leporids, sequence databases reflect biases toward those with greater clinical significance. Consequently, less is known about the ssDNA viral diversity in ticks.

In this study, we expand on the current knowledge of anellovirus diversity across different leporid hosts and ticks. Using a metagenomic approach, we identified full anellovirus genomes from leporid organ samples. For all samples, we determined the complete mitochondrial genomes of the four leporid samples and two associated tick samples.

## 2. Materials and Methods

### 2.1. Sample Collection

Two leporids that were hunter-harvested (2023) and two that were taken into a wildlife rescue center and subsequently died due to unknown causes (2024) were necropsied (Table 1). From these deceased leporids, we sampled the liver, spleen, and kidney. On the two hunter-harvested animals, we found ticks on their ears, which were sampled (Table 1). All samples were stored at −20 °C before processing.

### 2.2. DNA Extraction and Circular DNA Amplification

DNA was extracted from each sample type as follows. Organ samples (0.5 cm^3^) were each homogenized in 600 µL of SM buffer. Ticks were removed off the ear (n = 5) tissue and pooled per sample, and to this, 450 µL SM buffer and 50 µL Proteinase K were added and incubated at 55 °C for 2–4 h. The tick samples were then homogenized using a pestle in a 1.7 mL tube. All samples were centrifuged for 2 min at 10,000 rpm, and 200 µL of supernatant was used for nucleic acid extractions using the High Pure Viral Nucleic Acid Kit (Roche Diagnostics, Indianapolis, IN, USA). To amplify circular DNA molecules, 1 µL of DNA was used in a rolling-circle amplification (RCA) reaction with a phi29 DNA Polymerase Kit (Watchmaker Genomics, Boulder, CO, USA).

### 2.3. Illumina Library Preparation and Sequencing

To prepare samples for sequencing, 15 µL of the extracted viral DNA was combined with 15 µL of the RCA product. Organ samples from the same individual were pooled for sequencing. An Illumina DNA LP (M) Tag 96 sample preparation kit was used to prepare DNA high-throughput sequencing libraries (Illumina Inc., San Diego, CA, USA). The libraries were sequenced at Psomagen, Rockville, MD, USA, on an Illumina NovaSeq X Plus platform. Raw reads were trimmed using Trimmomatic v0.39 [33], followed by de novo assembly of paired-end reads into contigs using MEGAHIT v1.2.9 [34]. All contigs were analyzed for viral-like and mitochondrial sequences using DIAMOND BLASTx v2.1.8 [35] against a viral RefSeq protein and mitochondrial sequence database (Release 220). De novo assembled contigs were determined to be circular based on terminal redundancy.

### 2.4. Anellovirus Sequence Analysis

The anellovirus genomes were annotated with ORFfinder and checked manually in Geneious Prime 2023.2 (Dotmatics, Boston, MA, USA). A dataset of the anelloviruses obtained from this study coupled with 15 previously determined genomes from leporids was assembled. From these, we extracted the three coding regions (ORF1, ORF2, and ORF3) for downstream analysis. Pairwise identities of both the nucleotide and amino acid sequences were determined using SDT v1.2 [36].

An ORF1 dataset of the leporid-infecting anelloviruses, together with those of a rodent-infecting anellovirus (KJ194617) and a felid-infecting anellovirus (MT538139), as outgroups was assembled. Their ORF1 amino acid sequences were aligned with MAFFT [37]. The maximum likelihood phylogenetic tree was generated with PhyML [38], with the WAG + G amino acid substitution model determined as the best fit model using ProtTest 3 [39]. Branches with less than 60% bootstrap support were collapsed using TreeGraph 2.15 [40].

An alignment of the 19 anellovirus genomes was generated using MAFFT [37]. This alignment was then used to infer recombination events using RDP5 version 57 [41] with default parameters. Recombination events were considered credible when they showed >3 different recombination detection methods implemented in RDP5, comprising RDP (R), GENECONV (G), Maxchi (M), Chimaera (C), SiSscan (S), 3Seq (Q), *p*-value of <0.05, and phylogenetic support for recombination.

### 2.5. Mitochondrial Sequence Analyses

The mitochondrial genomes were annotated using MITOS [42]. In order to verify and, in some cases, determine the species, we compiled a dataset of mitochondrial sequences from the Barcode of Life Data Systems (BOLD) database [43,44] of representative Leporidae family and members of the *Dermacentor* genus (for tick identification). We undertook a phylogenetic analysis of the cytochrome c oxidase subunit 1 *(cox1*) gene region given that a larger number of sequences were available for reference species. These datasets were aligned using MAFFT [37]. Maximum likelihood phylogenetic trees were inferred using PhyML [38] using the best fit nucleotide substitution model GTR + G + I determined using jModelTest [45] for both datasets. Branches with less than 60% bootstrap support were collapsed using TreeGraph 2.15 [40]. The leporid *cox1* gene phylogenetic tree was rooted with the sequence of an annamite striped rabbit (Nesolagus timminsi, MW539689), whereas the one for the ticks was rooted with the sequence of a brown ear tick (Rhipicephalus appendiculatus, MT430988).

### 2.6. Tick Morphological Analyses

Twelve ticks, all nymphs, were observed with the help of a stereomicroscope (10–70×) and a compound microscope (100–200×) and identified to species using the key from Brinton, et al. [46] (Supplementary Figure S1).

## 3. Results and Discussion

### 3.1. Leporidae and Dermacentor Species Identification

We determined the complete mitochondrial genomes of these four leporids and the ticks that were found feeding on two of these leporids (sample # AZLag1 and AZLag2). The leporid mitochondrial genomes range from 16834 to 16882 nts in length, and these have been deposited in GenBank under accession numbers PQ664582-PQ664585. The raw reads mapping to these genomes have been deposited under BioProject # PRJNA1033669; BioSamples # SAMN45063108-SAMN45063111 and SRA #s SRR31517776-SRR31517779.

Two of the leporid mitochondrial genomes (sample # AZLag1 and AZLag2) share 99.7% pairwise identity. A BLASTn analysis of the four leporid mitochondrial genomes reveals that those from AZLag1 and AZLag2 share 97.94% identity with 97% query coverage and AZLag5 shares 97.87% with 98% query coverage with that of *Lepus alleni* (ON456168). The AZLag7 mitochondrial genome shares 86.35% with 98% query coverage with that of *Lepus tibetanus* (MN539746). A BLASTn analysis of the *cox1* gene shows that AZLag1 and AZLag2 share 99.55% identity and AZLag5 shares 99.22% identity with that of *Lepus californicus* (KJ397614), whereas that of AZLag7 shared 85.16% identity with that of *Lepus tibetanus* (MN539746).

**Figure 2:**
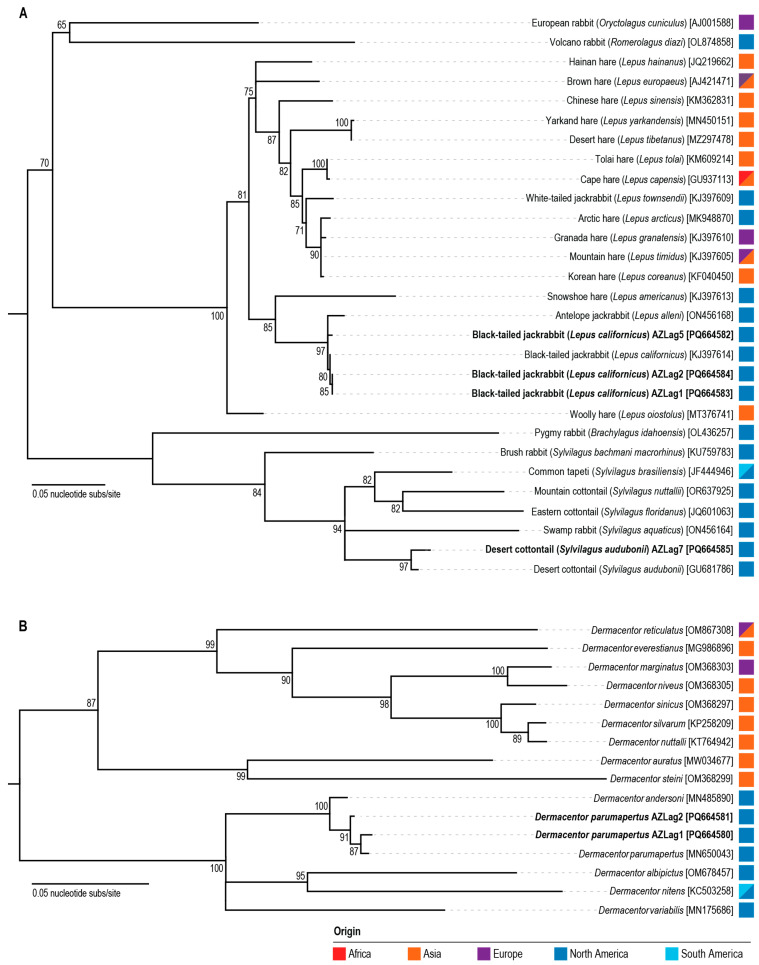
Leporid and tick host phylogenetic trees of *cox1* uncover biogeographical relationships. The estimated geographical range of each species is illustrated by the color-coded continent of origin. (**A**) The maximum likelihood phylogenetic tree reveals leporid evolutionary relationships. The leporid phylogeny is informed by partial *cox1* sequences from mitochondrial DNA genomes available in GenBank. (**B**) The maximum likelihood phylogenetic tree reveals tick evolutionary relationships in the *Dermacentor* genus. The tick phylogeny is informed by partial *cox1* sequences from mitochondrial DNA genomes available in GenBank and the BOLD database.

Phylogenetic analyses of *cox1* gene reveal that the four leporid sequences cluster together with those from other reference species (Figure 2A). The *Lepus* clade has the cox1 gene sequences from leporid samples AZLag1, AZLag2, and AZLag5 cluster most closely with that of a black-tailed jackrabbit (*Lepus californicus*) (Figure 2A), and the *cox1* gene sequence of AZLag7 is most closely related to the desert cottontail (*Sylvilagus audubonii*), aligning with the visual identifications.

To better understand our analyses of leporid phylogeny and biogeography, we reviewed earlier reports on their evolution. There are ~11 genera and ~60 species within the family Leporidae, and several factors continue to challenge the previously established taxonomies [47]. Leporids have a nearly global distribution, with varying levels of geographic isolation among taxa. However, *Lepus* is the only taxon with an almost cosmopolitan distribution [48]. The genus *Lepus* is also the most difficult to characterize due to its widespread range. According to biogeographical reports, intercontinental divergence events likely facilitated speciation events. Cytogenetic and mtDNA evidence suggest that the *Lepus* lineage originated in North America, before dispersing to Eurasia and Africa about 11.8 million years ago [48,49]. The development of vast temperate grasslands and land bridges likely promoted their intercontinental dispersal, causing *Lepus* to become the most species-rich leporid, with ~26 documented species [47]. In addition, mtDNA introgressions have been observed between arctic and temperate species, further supporting cross-hybridizations [50]. Within the *Lepus* clade in Figure 2A, the closest relative of AZLag1, AZLag2, and AZLag5 is the black-tailed jackrabbit (*Lepus californicus*), aligning with earlier reports on *Lepus* speciation in the western United States [47]. The closest relatives to AZLag7, the desert cottontail (*Sylvilagus audubonii*) and related *Sylvilagus* members, are also found in similar regions in the western United States [48,49].

The two tick mitochondrial genomes are 14,847 nts and 14,848 nts in length, and these have been deposited in GenBank under accession numbers PQ664580–PQ664581. The raw reads mapping to these genomes have been deposited under BioProject # PRJNA1033669; BioSamples # SAMN45063112-SAMN45063113 and SRA #s SRR31517774-SRR31517775. These two genomes share 99.27% pairwise identity and, when compared to sequences in GenBank using BLASTn, share 97.32% identity with 94% query coverage with the mitochondrial sequence of *Dermacentor andersoni* (MN485890). BLASTn analyses of the mitochondrial region *cox1* revealed that they are part of the family Ixodidae, genus *Dermacentor*. A maximum likelihood phylogenetic tree of *cox1* together with representative species in the genus *Dermacentor* shows that the ticks, recovered from leporid samples AZLag1 and AZLag2, are nested together in one clade between *D. andersoni* and *D. parumapertus* species (Figure 2B).

Prior work has highlighted the challenges in *Dermacentor* taxonomy due to hybridizations and introgressions within the genus [51]. These factors have complicated the evaluation of genetic differences at the species level. To more accurately identify the tick species, we explored their morphologies, geographic ranges, and host associations.

Morphological evaluation suggests that the recovered ticks are more closely related to *D. parumapertus*. Based on microscopy-generated images, the ticks (in the nymph stage) display features more similar to *D. parumapertus*, as opposed to *D. andersoni*. The external spur on coxa I is moderately small and does not extend past the anterior margin of coxa II, while the internal spur is indistinct. In addition, the larger goblet cells surrounding the macula on the spiracular plates are themselves surrounded by much smaller goblet cells rather than forming a continuous ring (Supplementary Figure S1). These observations are consistent with prior reports on the morphology of *D. parumapertus* [46] nymphs.

An analysis of the *Dermacentor* geographic range and host associations further supports the species identification of *D. parumapertus*. Within Arizona, the CDC range maps indicate that the *D. andersoni* species are restricted to the northern region of the state [52]. Although similar maps are not available for *D. parumapertus*, recent studies (on the bacterial pathogen *Rickettsia parkeri*) described the presence of *D. parumapertus* ticks in southern Arizona [53,54]. Their described range is consistent with our leporid sampling location in Tucson, Arizona. Between the two species, *D. andersoni* and *D. parumapertus* do not appear to be reciprocally monophyletic, which is evidenced by mito-nuclear discordance, hybridization, and introgression events [55]. Both species have been observed on the same hosts with sympatric distributions. Notably, *D. parumapertus* adult ticks are specialists that primarily parasitize black-tailed jackrabbits, whereas *D. andersoni* ticks are generalists that feed on a wider range of mammals. In summary, our tick report relies on mitochondrial phylogeny (Figure 2B), as well as other tick characteristics, including their morphology, geographic range, and host associations. We identify the ticks from this report as likely members of the species *D. parumapertus*, while recognizing the present uncertainties associated with the *Dermacentor* taxonomy.

### 3.2. Identification of Anelloviruses

**Figure 3:**
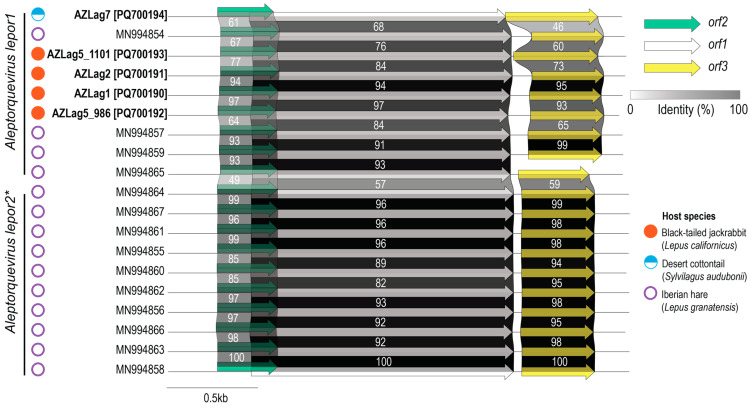
Genome organization of leporid anelloviruses. The five leporid genomes from this study share a similar genome organization with the previous ones identified from Iberian hares [22]. Genome organization and ORF1, ORF2, and ORF3 protein sequence comparison of the leporid-infecting anelloviruses generated using Clinker [56]. * Putative species name not officially classified.

From the four leporid samples, five anellovirus genomes were identified (Figure 3). These anellovirus genomes range from 2486 to 2559 nts in length (Figure 3). The five genomes have been deposited in GenBank under accession numbers PQ700190–PQ700194. The raw reads mapping to these genomes have been deposited under BioProject # PRJNA1190968; BioSamples # SAMN45063108-SAMN45063111 and SRA #s SRR31518301-SRR31518304. All the leporid anelloviruses share a similar genome organization (Figure 3). The lengths of *orf1*, *orf2*, and *orf3* of these viruses are 1407–1419 nts, 306–321 nts, and 393–507 nts, respectively. All five genomes have a GC-rich domain of ~60 nts in the untranslated region between *orf3* and *orf2*. No anelloviruses were identified in the ticks found feeding on the black-tailed jackrabbits.

**Figure 4:**
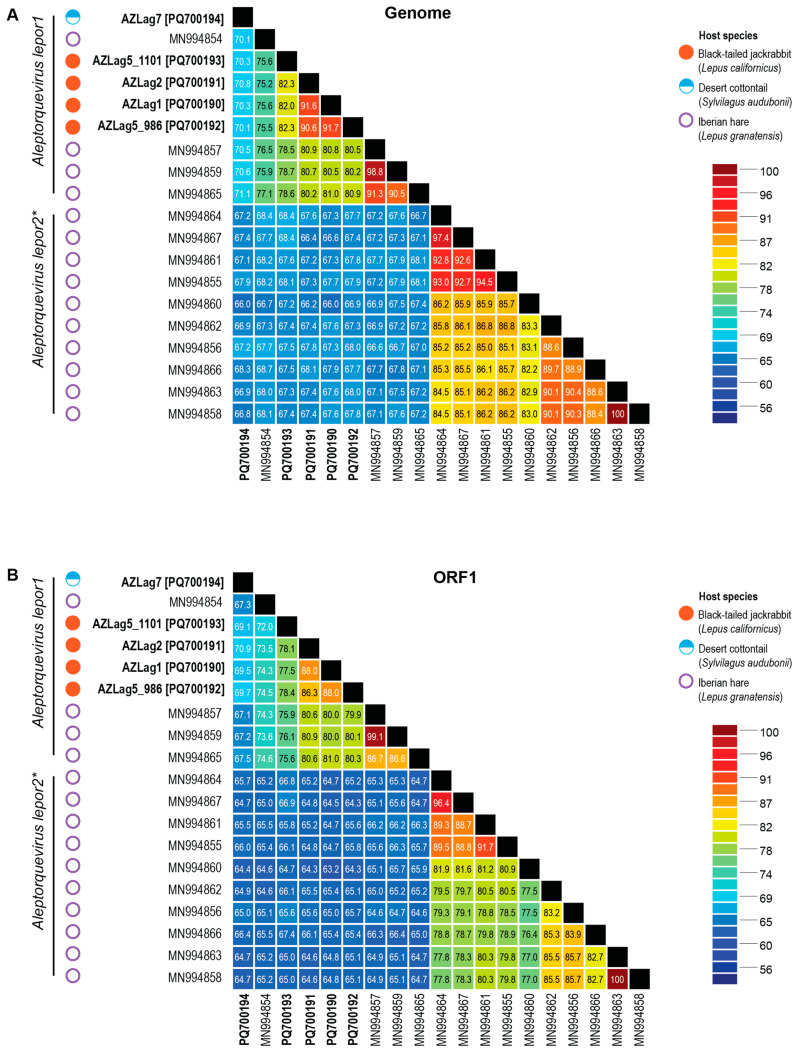
Pairwise identity matrix of the *orf1* gene sequence. (**A**) Pairwise identity matrix of the genomes of leporid-infecting anelloviruses. (**B**) *orf1* nucleotide sequences’ pairwise identity matrix of leporid-infecting anelloviruses. The pairwise identities were determined using SDT v1.2 [36]. * unassigned species.

In one black-tailed jackrabbit sample (sample # AZLag5; PQ664582), we identified two distinct anellovirus genomes (PQ700192 and PQ700193) that share 82.3% genome-wide pairwise identity. All genomes from black-tailed jackrabbits share 75.2–91.7% genome-wide identity with each other and 70.1–70.8% with that from a desert cottontail (Figure 4).

A pairwise analysis of the *orf1* gene, which is the most conserved region (encoding the capsid protein), across all anelloviruses, shows that the leporid anelloviruses fall into two clusters. The desert cottontail-infecting anellovirus from sample AZLag7 (PQ700194) is the most divergent of the five anelloviruses identified in this study and shares 69.1–70.9% *orf1* nucleotide pairwise identity with the other four anelloviruses from the black-tailed jackrabbit samples, AZLag1 (PQ700190), AZLag2 (PQ700191), and AZLag5 (PQ700192-PQ700193). (Figure 4). The *orf1* of AZLag5_986 (PQ700192) is most similar to AZLag1 (PQ700190), with 88% nucleotide pairwise identity.

**Figure 5:**
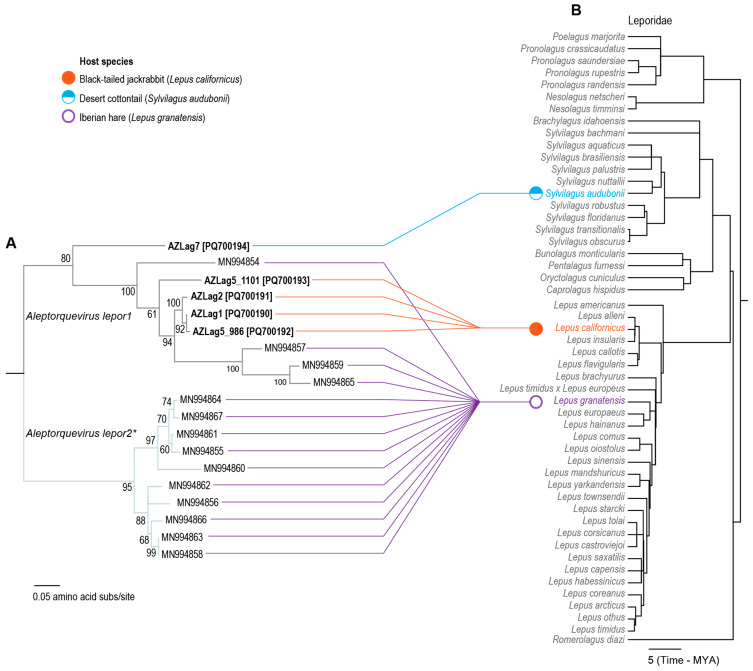
Tanglegram of the phylogenies of the ORF1 protein sequences of leporid-infecting anelloviruses and members of the Leporidae family. (**A**) Maximum likelihood phylogenetic tree of the ORF1 protein sequences of leporid anelloviruses. (**B**) Phylogeny of the members of the Leporidae family generated using TimeTree v5 [57]. * Putative species name not officially classified.

A phylogenetic analysis of the ORF1 protein (Figure 5) coupled with the pairwise identities (Figure 4) reveals that all of the leporid-infecting anelloviruses are part of the genus *Aleptorquevirus*. Within this genus, there is currently only one classified species, *Aleptorquevirus lepor1* [1], with isolate Lag01_EL_Anello4 (MN994854) from Iberian hares [22] as a representative. According to the *Anelloviridae* family taxonomy report, the species cutoff is 69% *orf1* pairwise nucleotide similarity [1]. Therefore, the five anelloviruses described here belong to the species *Aleptorquevirus lepor1*, with the desert cottontail-infecting anellovirus (sample # AZLag7; PQ700194) being the most divergent member of this species, and phylogenetically, it is basal to this clade (Figure 5).

Ten Iberian hare-infecting anelloviruses (MN994855, MN994856, MN994858, MN994860, MN994861, MN994862, MN994863, MN994864, MN994866, and MN994867) share <69% ORF1 pairwise identity with members of the species *Aleptorquevirus lepor1* (Figure 4) and thus represent members of a second species (*Aleptorquevirus lepor2*) within the genus *Aleptorquevirus*. This is also supported by the ORF1 amino acid sequence phylogenetic analysis (Figure 5). Overall, ORF1 of the leprid-derived anelloviruses across the two species (*Aleptorquevirus lepor1* and *Aleptorquevirus lepor2*) share 56.2–100% pairwise amino acid identity.

The leporid-infecting anelloviruses in the clade of the phylogenetic tree representing *Aleptorquevirus lepor1* show more diversity than those in the species *Aleptorquevirus lepor2*, which are all from Iberian hares. Furthermore, within the *Aleptorquevirus lepor1* clade, three Iberian hare-infecting anelloviruses (MN994857, MN994859, and MN994865) form a distinct subclade sharing > 87.8% ORF1 amino acid sequence (Figure 4) identity.

The phylogenetic analysis of the ORF1 protein reveals that there are at least eleven lineages (with genomes sharing > 90% genome-wide pairwise identity) of anelloviruses circulating in Iberian hares and two lineages in black-tailed jackrabbits (Figure 5). The lineage of anellovirus in the desert cottontail is unique. *Aleptorquevirus* variants were likely present in the last common ancestors of the *Lepus* and *Sylvilagus* genera (Figure 5). Anellovirus studies targeting the taxa *Romerolagus*, *Nesolagus*, *Poelagus*, and *Pronolagus* in the Leporidae family and members of the Ochotonidae family which are part of the broader Lagomorpha order would help determine the taxa host specificity of leporid- and ochotonid-infecting anelloviruses and any evolutionary signal of co-speciation with their hosts.

The ORF2 proteins of all the leprid-derived anelloviruses are homologous, sharing 46.7–100% amino acid pairwise identity. On the other hand, ORF3s are only homologous within species, with those of *Aleptorquevirus lepor1* sharing 52.8–99.2% and those of *Aleptorquevirus lepor2* sharing 61.9–100% amino acid identity.

**Figure 6:**
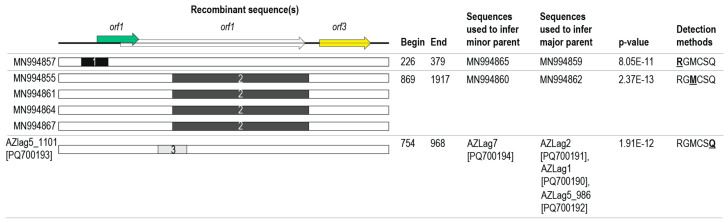
Detection of recombination events in the genomes of leporid-infecting anelloviruses. Summary of the three recombination events detected using RDP5 [41]. The position of the breakpoints are provided together with the putative major and minor parental sequences. The lowest *p*-value of the detection method is shown (bold underline text), and all detection methods that identified the recombination event are listed. Detection methods: RDP (R), GENECONV (G), Maxchi (M), Chimaera (C), SiSscan (S), and 3Seq (Q).

Recombination has been reported for all major clades of anelloviruses, with recombination breakpoint hotspots in the non-coding regions [5,8,58,59]. Our recombination analysis revealed three recombination events (Figure 6). The recombinant sequence MN994857 has a recombinant region of 153 nts derived from the MN994865-like sequence spanning the non-coding region and the part of the *orf1* gene, with the major parental sequence being the MN994859-like sequence. MN994855, MN994861, MN994867, MN994864 all share the same recombinant region of 1048 nts (spanning part of the *orf1* genes and a small part of the non-coding region) that is derived from MN994860-like sequences. The anellovirus recovered from AZLag5_1101 (PQ700193) has a recombinant region of 214 nts in the *orf1* gene that is derived from AZLag7-like sequences (Figure 6).

## 4. Conclusions

In this study, we characterized host genetic relationships from leporids and ticks and described novel leporid-infecting anelloviruses from the same system. Both leporids and ticks have complex origins, as individual species are adapted to diverse environments spanning multiple continents. Here, we determined the complete mitochondrial genome from four leporids and two ticks. We performed phylogenetic analyses on the *cox1* gene, given the limited availability of full mitochondrial sequences from representative species. Among the leporids, we identified three black-tailed jackrabbits (*L. californicus*) and one desert cottontail (*S. audubonii*). Notably, the biogeographical relationships highlight clades associated with a North American origin, whereas the neighboring species originate from distant continents. Combined with earlier reports, our results are consistent with *Lepus* intercontinental speciation hypotheses stemming from the global development of grasslands and land bridges ~11.8 MYA [48,49]. Among the ticks, we identified the two individuals as *D. parumapertus* members, which required additional morphological and ecological evaluation due to genetic hybridizations and introgressions in the genus [55].

The primary aim of this study was to assess the anelloviruses infecting leporids and associated with ticks feeding on these hosts. Prior work has emphasized viruses with great clinical significance as opposed to commensals with fewer, or a lack of, disease associations. The first leporid-infecting anelloviruses were described as viral coinfections with a myxomavirus-MYXV-Tol and/or a polyomavirus in Iberian hares (*L. granatensis*) [21]. Here, our high-throughput sequencing approaches facilitated the recovery of five complete anellovirus genomes from four leporid organ samples. These belong to the genus *Aleptorquevirus* and species *Aleptorquevirus lepor1*, which was initially established in the report found in [21]. Altogether, the ORF1 analyses and the leporid phylogeny provide evidence for a polyphyletic *Aleptorquevirus* origin. From the tick samples, we were unable to recover complete anellovirus genomes, yet we observed partial anellovirus sequences in the raw reads, which indicate they are taking up anelloviruses in the bloodmeal and may be able to move them between hosts. Overall, future viral metagenomic studies and host genetic studies encompassing more taxa would provide greater resolution in their evolutionary history, broadening the relationships shared between anelloviruses and their hosts.

## Figures and Tables

**Table 1:** Summary of sample information for all anellovirus genomes, leporid mitochondrial genomes, and tick mitochondrial genomes recovered in this study. These data include visually identified host species, sampling location, sample date, sample types, and accession numbers. * Hunter-harvested. # = number.

Sample ID	Visual Host Species	Sampling Location	Sample Date	Sample Types	Anellovirus Genome Accession #	Leporid Mitochondrial Genome Accession #	Tick Mitochondrial Genome Accession #
AZLag1 *	Black-tailed jackrabbit (*Lepus californicus*)	Tucson, AZ, USA	7 March 2023	Liver Kidney Ear with ticks	PQ700190	PQ664583	PQ664580
AZLag2 *	Black-tailed jackrabbit (*Lepus californicus*)	Tucson, AZ, USA	7 March 2023	Liver Kidney Ear with ticks	PQ700191	PQ664584	PQ664581
AZLag5	Black-tailed jackrabbit (*Lepus californicus*)	Buckeye, AZ, USA	7 August 2024	Liver Spleen	PQ700192, PQ700193	PQ664582	-
AZLag7	Desert cottontail (*Sylvilagus audubonii*)	Glendale, AZ, USA	28 August 2024	Liver Spleen	PQ700194	PQ664585	-

## Data Availability

The leporid mitochondrial genomes have been deposited in GenBank under accession numbers PQ664582–PQ664585. The raw reads mapping to these genomes have been deposited under BioProject # PRJNA1033669; BioSamples # SAMN45063108-SAMN45063111 and SRA #s SRR31517776-SRR31517779. The two tick mitochondrial genomes have been deposited in GenBank under accession numbers PQ664580–PQ664581. The raw reads mapping to these genomes have been deposited under BioProject # PRJNA1033669; BioSamples # SAMN45063112-SAMN45063113 and SRA #s SRR31517774-SRR31517775. The five genomes have been deposited in GenBank under accession numbers PQ700190–PQ700194. The raw reads mapping to these genomes have been deposited under BioProject # PRJNA1190968; BioSamples # SAMN45063108-SAMN45063111 and SRA #s SRR31518301-SRR31518304.

## Supplementary Materials

The following supporting information can be downloaded at https://www.mdpi.com/article/10.3390/v17020280/s1, Supplementary Figure S1: Individual tick representing those from AZLag1 and 2 showing morphologies that support identification. (A) Dorsal view of a tick sample. (B) Ventral view of a tick sample. (C) The red arrows point to the external spur on coxa I (both sides), while the blue arrows point to the much smaller internal spur. (D) The orange arrow points to the spiracle. The larger goblet cells surrounding the macula on the spiracular plates are themselves surrounded by much smaller goblet cells rather than forming a continuous ring.
