# Supplementary figures and images for: Diverse Anelloviruses Identified in Leporids from Arizona (USA)
Source: Viruses. 2025 Feb 18;17(2):280. doi: 10.3390/v17020280 (PMC11860494; doi:10.3390/v17020280)

A

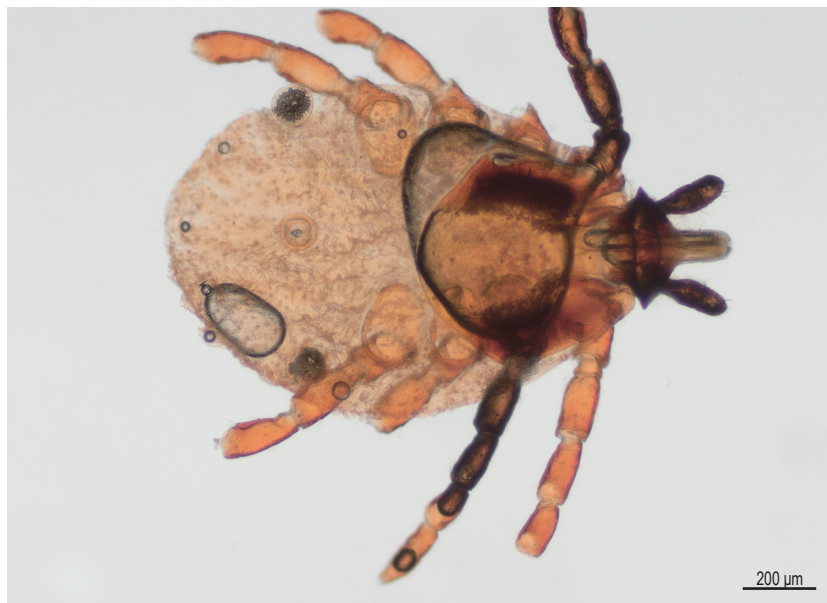

B

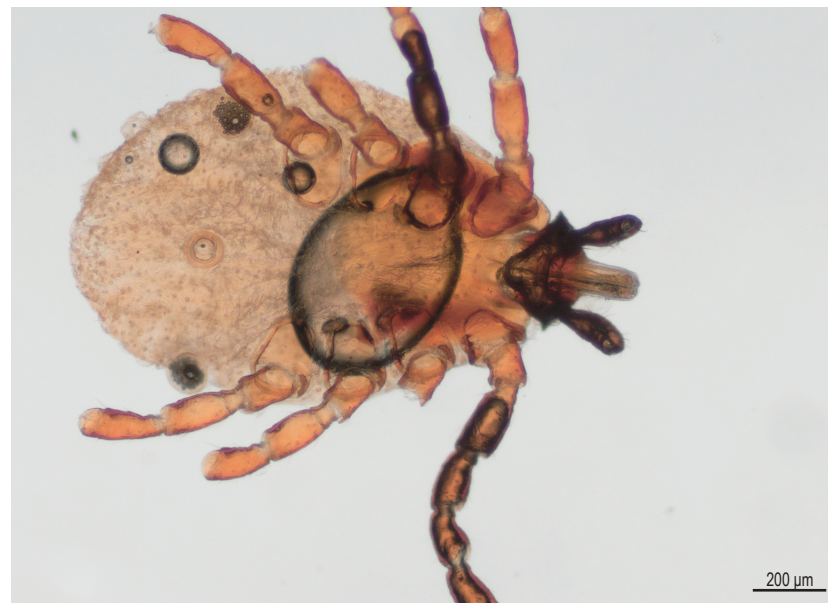

C

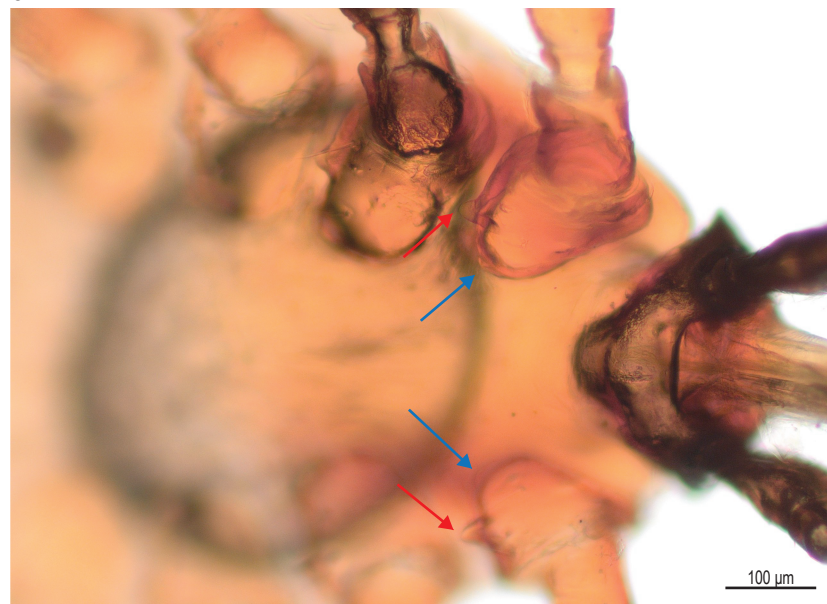

D

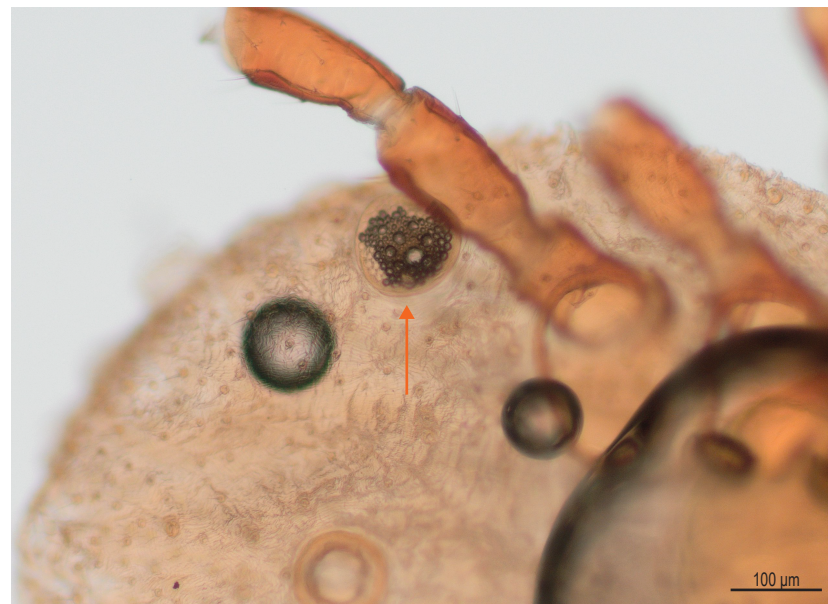

Supplement: Supplementary file 1 [file viruses-17-00280-s001.zip › viruses-3447173-supplementary.pdf]
